# DNA Methylation Signature of a Lifestyle‐based Resilience Index for Cognitive Health

**DOI:** 10.1002/alz70856_107284

**Published:** 2026-01-08

**Authors:** Lily Wang, Deirdre O'Shea, Wei Zhang, David Lukacsovich, Juan I Young, Lissette Gomez, Michael A. Schmidt, Eden R. Martin, Brian W Kunkle, X. Steven Chen, James E. Galvin

**Affiliations:** ^1^ Department of Public Health Sciences, University of Miami Miller School of Medicine, Miami, FL, USA; ^2^ University of Miami Miller School of Medicine, Boca Raton, FL, USA; ^3^ University of Miami Miller School of Medicine, Miami, FL, USA; ^4^ Dr. John T. Macdonald Foundation Department of Human Genetics, University of Miami Miller School of Medicine, Miami, FL, USA; ^5^ John P. Hussman Institute for Human Genomics, University of Miami Miller School of Medicine, Miami, FL, USA

## Abstract

**Background:**

Cognitive resilience (CR) contributes to the variability in risk for developing and progressing in Alzheimer's disease (AD) among individuals. Beyond genetics, recent studies highlight the critical role of lifestyle factors in enhancing CR and delaying cognitive decline. DNA methylation (DNAm), an epigenetic mechanism influenced by both genetic and environmental factors, including CR‐related lifestyle factors, offers a promising pathway for understanding the biology of CR.

**Method:**

We studied DNAm differences associated with the Resilience Index (RI), a composite measure of lifestyle factors, using blood samples from the Healthy Brain Initiative (HBI) cohort. Differential methylation analyses were conducted at both the individual CpG level and across genomic regions, using robust linear models and the comb‐p software. These analyses were adjusted for age, sex, APOE ε4, and immune cell compositions, with corrections for inflation and multiple testing.

**Result:**

Our analysis identified 19 CpGs and 24 differentially methylated regions significantly associated with the RI. The RI‐associated methylation changes are significantly enriched in pathways related to lipid metabolism, synaptic plasticity, and neuroinflammation, and highlight the connection between cardiovascular health and cognitive function. Furthermore, we developed a Methylation‐based Resilience Score (MRS) that successfully predicted future cognitive decline in an external dataset from the Alzheimer's Disease Neuroimaging Initiative (ADNI), even after accounting for age, sex, *APOE ε4*, years of education, baseline diagnosis, and baseline MMSE score.

**Conclusion:**

By identifying RI‐associated DNAm, our study provided an alternative approach to discovering future targets and treatment strategies for AD, complementary to the traditional approach of identifying disease‐associated variants directly. Our findings are particularly relevant for a better understanding of epigenetic architecture underlying cognitive resilience. Importantly, the significant association between baseline MRS and future cognitive decline demonstrated that DNAm could be a predictive marker for AD, laying the foundation for future studies on personalized AD prevention.

